# Internal control genes for quantitative RT-PCR expression analysis in mouse osteoblasts, osteoclasts and macrophages

**DOI:** 10.1186/1756-0500-4-410

**Published:** 2011-10-14

**Authors:** Alexandre S Stephens, Sebastien R Stephens, Nigel A Morrison

**Affiliations:** 1 School of Medical Science, Griffith University, Gold Coast, Queensland, Australia

## Abstract

**Background:**

Real-time quantitative RT-PCR (qPCR) is a powerful technique capable of accurately quantitating mRNA expression levels over a large dynamic range. This makes qPCR the most widely used method for studying quantitative gene expression. An important aspect of qPCR is selecting appropriate controls or normalization factors to account for any differences in starting cDNA quantities between samples during expression studies. Here, we report on the selection of a concise set of housekeeper genes for the accurate normalization of quantitative gene expression data in differentiating osteoblasts, osteoclasts and macrophages. We implemented the use of geNorm, an algorithm that determines the suitability of genes to function as housekeepers by assessing expression stabilities. We evaluated the expression stabilities of 18S, ACTB, B2M, GAPDH, HMBS and HPRT1 genes.

**Findings:**

Our analyses revealed that 18S and GAPDH were regulated during osteoblast differentiation and are not suitable for use as reference genes. The most stably expressed genes in osteoblasts were ACTB, HMBS and HPRT1 and their geometric average constitutes a suitable normalization factor upon which gene expression data can be normalized. In macrophages, 18S and GAPDH were the most variable genes while HMBS and B2M were the most stably expressed genes. The geometric average of HMBS and B2M expression levels forms a suitable normalization factor to account for potential differences in starting cDNA quantities during gene expression analysis in macrophages. The expression stabilities of the six candidate reference genes in osteoclasts were, on average, more variable than that observed in macrophages but slightly less variable than those seen in osteoblasts. The two most stably expressed genes in osteoclasts were HMBS and B2M and the genes displaying the greatest levels of variability were 18S and GAPDH. Notably, 18S and GAPDH were the two most variably expressed control genes in all three cell types. The geometric average of HMBS, B2M and ACTB creates an appropriate normalization factor for gene expression studies in osteoclasts.

**Conclusion:**

We have identified concise sets of genes suitable to use as normalization factors for quantitative real-time RT-PCR gene expression studies in osteoblasts, osteoclasts and macrophages.

## Background

The development of the skeleton and its maintenance during adulthood requires the stringent control of gene regulatory programs in response to physiological signals. These gene expression regulatory cascades operate in all the major cells of bone including the bone forming osteoblasts and the bone degrading osteoclasts [1]. Recent research on osteoblasts and osteoclasts has commonly used quantitative gene expression analysis to investigate the regulatory mechanisms which operate within them (e.g. [2-7]).

Real-time quantitative reverse transcriptase-PCR (qPCR) is a powerful technique that can accurately detect low abundance mRNAs [8]. qPCR is fast, efficient, does not require post-PCR processing and functions over a large dynamic range of starting cDNA quantities [9,10]. These qualities have made qPCR the method of choice for accurately quantifying gene expression levels [10]. However, qPCR can suffer from certain limitations which can lead to substantial variability in expression measures [11]. One of the most important issues relates to the selection of appropriate normalization factors to account for any errors and differences generated through the multi-step process involved in producing cDNA [12]. Various strategies have been implemented for data normalization including normalizing to cell numbers, genomic DNA and RNA input [12]. Each of these methods suffers from limitations and their use in some circumstances could lead to inaccurate data normalization [12]. For example, normalizing to RNA can be problematic if limited amounts are available for quantitation. In addition, total RNA is predominantly composed of rRNA and is not always reflective of mRNA content due to imbalances between rRNA and mRNA levels [13]. Furthermore, use of RNA content, genomic DNA or cell numbers does not take into consideration the efficiency of reverse transcriptase during cDNA synthesis reactions.

The most common method for data normalization involves the use of internal control (housekeeper) genes [12]. Housekeeper genes are presumed to be constitutively expressed and should display stable expression under a variety of experimental conditions. However, there is mounting evidence to suggest that the expression of internal reference genes may vary significantly under different experimental conditions opening the possibility that erroneous information could be generated if data normalization is based on genes that themselves are regulated [14-16]. An approach to circumvent the problems associated with potentially regulated internal control genes is to assess the validity of candidate reference genes in specific experimental contexts.

Here, we have taken into consideration the potential problems associated with using non-validated control genes for quantitative gene expression analyses and have set out to identify suitable reference genes for the normalization of qPCR gene expression data in mouse osteoblasts, osteoclasts and macrophages. We evaluated a set of six reference genes: 18S, ACTB, B2M, GAPDH, HMBS and HPRT1. These genes were selected as they have been commonly used as internal controls for quantitative gene expression analyses in published studies (e.g [17-20]) and encode for proteins or products that belong to distinct functional classes reducing the potential that the genes might be co-regulated. The geNorm algorithm [21] was implemented to assess the expression stabilities of the six candidate genes and we have identified concise sets of genes that constitute suitable normalization factors for gene expression studies in osteoblasts, osteoclasts and macrophages.

## Results

### Osteoblast, osteoclast and macrophage differentiation

**Figure 1:**
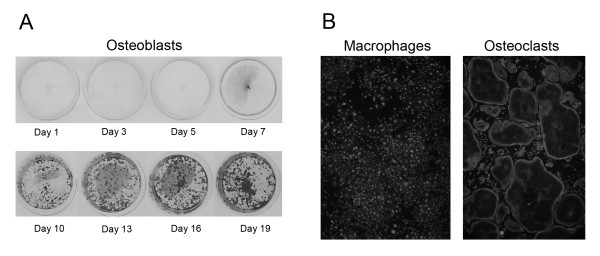
**Osteoblast, osteoclast and macrophage differentiation**. (A) MC3T3-E1 cells were seeded in 24-well culture plates and induced to differentiate into osteoblasts via the addition of medium containing 50 μg/ml ascorbic acid and 10 mM β-glycerophosphate. Cells were harvested at various time points throughout differentiation for gene expression studies. The figure represents MC3T3-E1 cells at various stages during the developmental process. The cells were stained with Alizarin Red S which is retained by mineralized extracellular matrix. (B) Bone marrow derived monocytes were seeded in 24-well culture plates and induced to differentiate into macrophages or osteoclasts via the addition of M-CSF or M-CSF + RANKL respectively. The figure displays representative photos of the macrophages and osteoclasts used in the study. The cells were stained with rhodamine phalloidin (F-actin stain) and DAPI (nucleic acid stain).

Osteoblastic cells were generated through the ascorbic acid-induced differentiation of MC3T3-E1 preosteoblasts. Differentiating cells were harvested for RNA extraction at various stages during the process. Figure 1A displays representative photos of osteoblasts stained with alizarin red S at the various time points used for the gene expression analyses. The abundance of alizarin red S staining observed in late stage osteoblasts (days 16 and 19) indicated that the MC3T3-E1 cells differentiated into mature osteoblasts which produced extensively mineralized extracellular matrix. Macrophages and osteoclasts were differentiated from primary mouse bone marrow monocytes treated with macrophage colony stimulating factor (M-CSF) and M-CSF + Receptor activator of nuclear factor kappa-B ligand (RANKL) respectively. Large, multinucleated cells with defined actin rings were clearly evident by four days of M-CSF + RANKL treatment indicating successful osteoclast formation. Figure 1B displays representative photos of the macrophages and osteoclasts used in the study.

### PCR efficiencies (E)

To identify reliable reference genes for qPCR expression analysis in osteoblasts, macrophages and osteoclasts, we evaluated the relative expression of six candidate genes: 18S, ACTB, B2M, GAPDH, HMBS and HPRT1 (table 1). We implemented the use of the ΔCT (difference between cycle threshold (CT) values) method to generate relative expression data via the relationship (E+1)^-ΔCT^. Cycle threshold is defined as the cycle number at which the fluorescence intensity of a product during a PCR run exceeds the fluorescence intensity threshold level. The threshold level is commonly set just above background fluorescence intensity at a level that falls within the exponential phase of the amplification curve. In order for the ΔCT method to provide the most accurate relative gene expression data, the PCR efficiencies of the genes from which the ΔCTs are calculated need to be equal. To this end, we used a 3-fold dilution series covering a three to four log dynamic range to generate standard curves. PCR efficiencies were calculated for each primer pairs (table 2) and ranged from 94-96%. The R^2 ^values for the standard curves of the candidate genes were ≥ 0.99 (table 2) reflecting high precision. PCR efficiency is a function of standard curve regression slope (b) and the comparison of the multiple regression slopes via the global F-test revealed there were no significant differences between the candidate genes (p-value = 0.985) indicating equal PCR efficiencies.

**Table 1:** Candidate reference gene symbols, names, functions and Genbank accession numbers

Symbol	Gene name	function	Accession
18S	18s Ribosomal RNA	Eukaryotic small ribosomal subunit	[Genbank:NR_003278]
ACTB	β-actin	Cytoskeletal structural protein	[Genbank:NM_007393]
B2M	β-2-microglobulin	Beta-chain of MHC class I molecules	[Genbank:NM_009735]
GAPDH	Glyceraldehyde-3- phosphate dehydrogenase	Oxidoreductase in glycolysis and gluconeogenesis	[Genbank:NM_008084]
HMBS	Hydroxymethylbilane synthase	Heme synthesis, porphyrin metabolism	[Genbank:NM_013551]
HPRT1	Hypoxanthine guanine phosphoribosyl transferase 1	Purine synthesis through the purine salvage pathway	[Genbank:NM_013556]

**Table 2:** Candidate reference gene PCR primer sequences (5'-3'), amplicon sizes, PCR efficiencies and standard curve regression coefficients

Symbol	Forward primer	Reverse primer	Amplicon size (bp)	PCR efficiency (E)	Regression coefficient (R^2^)
18S	CTTAGAGGGACAAGTGGCG	ACGCTGAGCCAGTCAGTGTA	107	95%	0.9947
ACTB	CTCTGGCTCCTAGCACCATGAAGA	GTAAAACGCAGCTCAGTAACAGTCCG	200	94%	0.9975
B2M	CTGCTACGTAACACAGTTCCACCC	CATGATGCTTGATCACATGTCTCG	241	96%	0.9986
GAPDH	ACAGTCCATGCCATCACTGCC	GCCTGCTTCACCACCTTCTTG	266	95%	0.9981
HMBS	GAGTCTAGATGGCTCAGATAGCATGC	CCTACAGACCAGTTAGCGCACATC	250	94%	0.9962
HPRT1	GAGGAGTCCTGTTGATGTTGCCAG	GGCTGGCCTATAGGCTCATAGTGC	173	95%	0.9943

### Expression profiling of candidate reference genes

**Figure 2:**
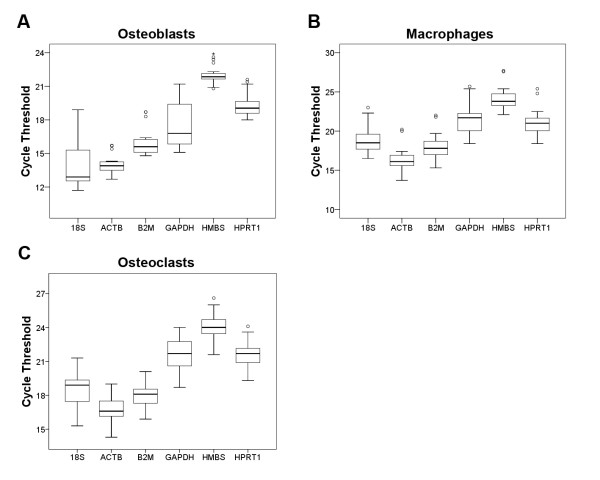
**Distribution of qPCR cycle threshold values for the candidate reference genes**. The expression of 18S, ACTB, B2M, GAPDH, HMBS and HPRT1 candidate internal control genes in osteoblasts (A), macrophages (B) and osteoclasts (C) are presented as box and whisker plots. Circles and asterisks represent outliers.

We investigated the expression of the six candidate reference genes in differentiating osteoblasts, macrophages and osteoclasts using a SYBR green based qPCR assay. The amplification CTs varied between the candidate genes and figure 2 shows their distributions. The distribution of the CT values provides a global representation of the variation in reference gene expression while also providing information on their relative abundances. More highly expressed genes are associated with lower CT values and the rank-order of most abundantly to least abundantly expressed genes in the three cell types were very similar. In macrophages and osteoclasts, the order of abundance from high to low was ACTB, B2M, 18S, GAPDH, HPRT1 and HMBS. In osteoblasts, the rank-order of abundance was 18S, ACTB, B2M, GAPDH, HPRT1 and HMBS.

### geNorm stability analysis

In order to identify suitable internal control genes for the normalization of quantitative gene expression data in mouse osteoblasts, osteoclasts and macrophages, we assessed the expression stabilities of six candidate genes using geNorm. The premise of geNorm is to determine the most stably expressed genes by calculating the average pair-wise variation in the log_2 _transformed expression ratios between one particular candidate gene and all other candidate genes. This process is repeated for all candidate genes and the most stable genes are those with the smallest average pair-wise variation. Table 3 shows the ranking of the candidate genes according to their expression stabilities. The stability values represent the average standard deviation of the CT differences between each gene and all other genes. In osteoblasts, stability values ranged from 0.776 to 1.240 with 18S being the least stably expressed and B2M displaying the greatest expression stability. The stability values in macrophages and osteoclasts were slightly smaller compared to osteoblasts indicating greater overall expression stability of the panel of genes. The most stably expressed gene in macrophages and osteoclasts was HMBS whereas the most variable genes were 18S for macrophages and GAPDH for osteoclasts.

**Table 3:** Expression stability values of candidate reference genes according to geNorm

Osteoblasts		Macrophages		Osteoclasts	
**Gene**	**Stability**	**Gene**	**Stability**	**Gene**	**Stability**

B2M	0.776	HMBS	0.502	HMBS	0.692
HPRT1	0.820	B2M	0.506	B2M	0.743
HMBS	0.834	HPRT1	0.557	ACTB	0.762
ACTB	0.882	ACTB	0.586	HPRT1	0.781
GAPDH	1.219	GAPDH	0.674	18S	0.921
18S	1.240	18S	0.691	GAPDH	0.925

**Figure 3:**
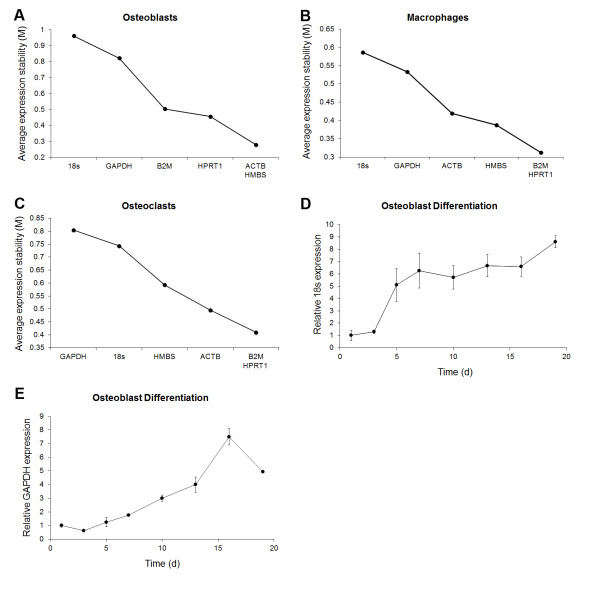
**Average expression stability values in osteoblasts, macrophages and osteoclasts and expression profiling of 18S and GAPDH during osteoblast differentiation**. qPCR gene expression analyses were carried out on cDNA derived from differentiating osteoblasts, macrophages and osteoclasts. Gene expression data were converted to relative expression using the relationship (E+1)^-ΔCT ^and the data were analyzed using the geNorm algorithm to identify the rank-order of gene expression stabilities in osteoblasts (A), macrophages (B) and osteoclasts (C). Transcript profiling of 18S and GAPDH throughout osteoblast differentiation (D-E). Gene expression data were normalized to the geometric average of ACTB, B2M, HMBS and HPRT1 and are expressed as relative expression to day 1. Data represents mean ± SEM.

Figure 3 displays the average expression stability values (M) of all control genes and of the remaining control genes after each sequential removal of the most variable gene for the different cell types. The figure also displays the rank-order of gene expression stabilities. For each of the groups, M displayed a consistent decline with each removal of the most variable gene. Notably, the rank-orders of stabilities in figure 3A-C are slightly different to that listed in table 3. This reflects the different methods in which the stability values were determined: in table 3, expression stability values were calculated using data from all control genes and the expression stability values for each individual gene was listed; in the figures, the rank-order of stabilities was determined by calculating the average expression stability values of all remaining genes after the sequential removal of the most variable genes. Ultimately, the figure-based method identifies the subset of genes that are the most correlated and have the highest expression stability values.

Osteoblasts displayed the highest initial M reflecting an overall increased variability compared to macrophages and osteoclasts. Notably, the osteoblast M dropped dramatically after the removal of the two most variable genes, 18S and GAPDH, and suggested these genes were highly variable or potentially regulated. To explore these possibilities, we plotted the relative expression of 18S and GAPDH throughout osteoblast differentiation using a normalization factor derived from the geometric average of ACTB, B2M, HMBS and HPRT1 (figures 3D-E). The plots showed that 18S and GAPDH expression increased over time indicating the genes were regulated throughout osteoblast differentiation. As such, both 18S and GAPDH would be unsuitable to use as internal control genes in osteoblasts. Macrophage and osteoclast samples also displayed considerable decreases in M after the sequential removal of the two most variable genes (figures 3B-C) which were also 18S and GAPDH. However, unlike osteoblasts, 18S and GAPDH expression levels in macrophages and osteoclasts did not change dramatically over time and the fold change in expression at days 2-5 relative to day 1 stayed within 2-fold (data not shown). These results suggested that the heightened variability of 18S and GAPDH genes in macrophage and osteoclast samples was not due to differential expression over time but a consequence of broader basal expression levels.

**Figure 4:**
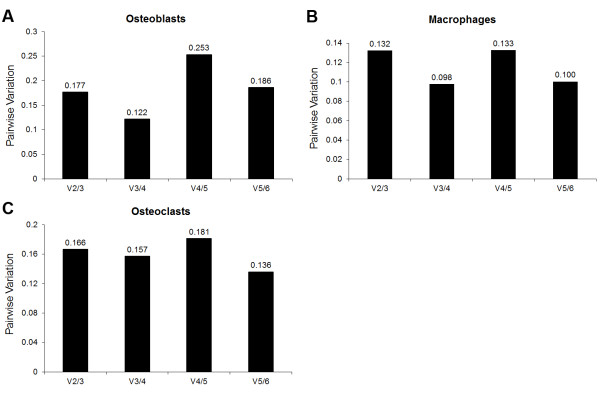
**Determination of the optimal number of control genes for the normalization of quantitative gene expression data in osteoblasts, macrophages and osteoclasts**. Relative gene expression data were assessed by geNorm and the pairwise variations between consecutive normalization factors were calculated. V(X)/(Y) signify the variance between consecutive normalization factors. For example, V2/3 represents the variance between NF2 (geometric average of two most stably expressed genes) and NF3 (geometric average of three most stably expressed genes).

The determination of the optimal number of housekeeper genes for each of the cell types is shown in figure 4. The figure displays the pairwise variation between the preceding normalization factor (NF_n_) and the current normalization factor (NF_n+1_) which differs from NF_n _by including the next most stably expressed gene in the calculation of the normalization factor. Essentially, the point at which the inclusion of an additional reference gene in the calculation of the NF_n+1 _only imposes a marginal change compared to NF_n _determines the optimal number of genes required. Vandesompele *et al *[21] suggested this point was reached when the pairwise variation fell below 0.15. Based on this cut-off, the optimum number of housekeeper genes required for the normalization of qPCR data is three for osteoblasts, two for macrophages and six for osteoclasts. However, for osteoclasts, the pairwise variation between the third and fourth normalization factors was a borderline value of 0.157. Thus, the geometric average of the three most stably expressed genes would most likely be sufficient for use as a normalization factor. Based on the determination of the optimal number of housekeeper genes, the constituents of the normalization factors for each cell type were determined. These genes consisted of ACTB, HMBS and HPRT1 in osteoblasts; B2M and HPRT1 in macrophages; and of ACTB, B2M and HPRT1 in osteoclasts.

## Discussion

We have assessed the expression of six candidate reference genes in osteoblasts, osteoclasts and macrophages and have identified the most suitable set of these genes to use for the accurate normalization of qPCR gene expression data in these cells. Our investigation implemented use of the geNorm algorithm which identifies appropriate housekeepers by determining which genes are the most stably expressed. The reliability and reproducibility of qPCR gene expression studies is highly dependent upon the selection of appropriate housekeeper genes or NFs to accurately adjust for differences in starting cDNA quantities between samples. Biological variation in the NF will inherently be transferred to gene expression data and thus the need to minimise this variation is important for producing reliable results. GeNorm provides a platform upon which to identify stably expressed genes and thus minimise the variation within NFs.

Our implementation of the geNorm algorithm evaluated the suitability of 18S, ACTB, B2M, GAPDH, HMBS and HPRT1 as potential reference genes. In osteoblasts, ACTB, HMBS, HPRT1 and B2M displayed the smallest amounts of expression variability (most stably expressed) and thus were flagged as being the most suitable for use in the calculation of a potential NF. The expression stabilities of ACTB, HMBS, HPRT1 and B2M ranged from 0.776 to 0.882 and indicated that the average standard deviation of the CT differences between each gene and all other (five) genes was less than one cycle. The magnitude of the expression variability attributable to factors other than biological sources such as pipetting errors and thermal cycler inconsistencies can be estimated by determining the variation in replicate samples. For our SYBR green based qPCR assay, this quantity was determined to be 0.243 cycles (data not shown). Not surprisingly, this quantity was smaller than the expression stabilities of ACTB, HMBS, HPRT1 and B2M and suggested that additional (biological) variability contributed to the expression stability values. Hypothetically, 0.243 would be the minimum gene expression stability value we could expect to obtain in the situation where all genes used in the calculations were perfectly correlated. Using this minimum value, we can estimate the contribution that biological variation makes towards the expression stability values by calculating the difference between the stability values and 0.243; thus for ACTB, HMBS, HPRT1 and B2M, biological variation accounted for between 0.533 to 0.639 cycles which represented 69-72% of the total stability values. GAPDH and 18S showed greater expression variability than ACTB, HMBS, HPRT1 and B2M and were regulated throughout differentiation. As such, these genes would not be suitable to use for the normalization of gene expression data in MC3T3-E1 osteoblasts. Accordingly, the determination of the optimal number of candidate genes to include in the calculation of the NF does not incorporate either GAPDH or 18S. Rather, geNorm indicated that a NF based on the geometric average of ACTB, HMBS and HPRT1 would be sufficient to control for differences in starting cDNA quantities between samples.

In osteoclasts, ACTB, B2M, HMBS and HPRT1 were also the most stably expressed genes leaving 18S and GAPDH as the most variably expressed. On average, the stability values of the candidate reference genes in osteoclasts were smaller in magnitude compared to those observed in osteoblasts indicating greater overall expression stabilities. The estimation of the contribution of biological variation towards the stability values of ACTB, B2M, HMBS and HPRT1 in osteoclasts ranged between 0.449 and 0.538 cycles representing 65-69% of the total stability values. The determination of the optimal number of control genes for the normalization of osteoclast gene expression data indicated that three genes were sufficient. These genes were ACTB, B2M and HPRT1 and their geometric average constitutes the NF.

The magnitudes of the stability values of the candidate internal control genes in differentiating macrophages were consistently the smallest out the of the three cell types tested. However, just like osteoblasts and osteoclasts, ACTB, B2M, HMBS and HPRT1 were again the most stably expressed genes. Biological variation accounted for between 52-59% of the total stability values derived for these genes. Analysis of the pair-wise variation between consecutive NFs revealed the geometric average of B2M and HPRT1 would be sufficient to constitute a normalization factor to account for any differences in starting cDNA quantities for gene expression studies in macrophages.

## Conclusion

We have investigated the expression stabilities of six candidate reference genes in osteoblasts, osteoclasts and macrophages. Our analysis has identified concise sets of genes that could be used for the accurate normalization of qPCR data in these cells. For osteoblasts, the geometric average of ACTB, HMBS and HPRT1 was determined to be a suitable combination of genes to constitute a NF for gene expression studies. In osteoclasts, the grouping of ACTB, B2M and HPRT1 were determined to form a reliable NF to normalize gene expression data. The combination of B2M and HPRT1 established a suitable NF for gene expression studies in macrophages.

## Materials and methods

### Tissue culture

MC3T3-E1 cells (sub clone 14) were maintained in Minimum Essential Medium (MEM) α Medium (Invitrogen) containing 10% Fetal Bovine Serum (Invitrogen), 1% Penicillin/Streptomycin solution (Invitrogen) and 1 mM Sodium Pyruvate (Invitrogen). For osteoblast differentiation, cells were seeded in 24-well culture plates at a density of 2.5 × 10^4 ^cells in a total volume of 0.5 ml of standard medium per well. 48 hours post-seeding, the medium was changed with fresh medium containing 50 μg/ml ascorbic acid (AA) and 10 mM beta-glycerophosphate (osteogenic medium). Medium was changed every 72 hours. For osteoblast differentiation time course, MC3T3-E1 cells were harvested 1, 3, 5, 7, 10, 13, 16 and 19 days post-addition of osteogenic medium. The number of samples per time point was three (total n = 24). Mouse bone marrow monocytes (BMMs) were prepared from bone marrow of 4 to 6 week-old C57BL/6 mice. Briefly, femurs and tibias were extracted from sacrificed mice and excess tissue was removed from the bones by scraping. The epiphyses were removed and the marrow cavities were flushed out with complete medium (Minimum Essential Medium (MEM) α Medium (Invitrogen) containing 10% Fetal Bovine Serum (Invitrogen), 1% Penicillin/Streptomycin solution (Invitrogen)) using a 26G needle. Bone marrow cells (BMCs) were recovered via centrifugation and seeded into culture vessels for 24 hours in complete medium containing 5 ng/ml M-CSF (Peprotech). Non-adherent BMCs were collected and seeded into culture vessels for a further 48 hours in complete medium containing 30 ng/ml M-CSF to generate BMMs. For macrophage and osteoclast differentiation, BMMs were seeded in 24-well culture plates at a density of 3 × 10^4 ^cells/well in a total volume 0.5 ml of complete medium containing 30 ng/ml M-CSF. Osteoclasts were generated via the addition of RANKL (Peprotech) to a final concentration of 35 ng/ml. A full medium change was performed on day 3 and cells were harvested on days 1, 2, 3, 4 and 5 post-initiation of differentiation for RNA extraction. A total of 20 samples (n = 4 per time point) for each macrophages and osteoclasts were used in the study. Procedures involving mice were approved by the animal ethics committee of Griffith University and the study had the approval of the ethics committee of Griffith University.

### Cell staining

MC3T3-E1 cells were stained with Alizarin Red S for detection of extracellular matrix mineralization. For staining, MC3T3-E1 cells were washed with one volume of PBS and fixed with 3.7% formaldehyde in PBS for 15 min. Fixed cells were washed twice with dH_2_O and incubated with 200 μl of 40 mM Alizarin Red S, pH 4.1 with gentle shaking for 20 min. The dye solution was removed and cells were washed four times with 1.5 ml dH_2_O with gentle shaking for 5 min per wash prior to photography. Mature macrophages and osteoclasts (day 5) were stained with rhodamine phalloidin (F-actin labelling, Invitrogen) and 4',6-diamidino-2-phenylindole, dihydrochloride (DAPI, nucleic acid stain, Invitrogen). For staining, cells were fixed with 3.7% formaldehyde in PBS for 15 min. The fixing solution was removed and cells were washed two times with PBS. The cells were then solubilized with the addition of 0.1% Triton X-100 solution in PBS for 10 min. The Triton X-100 solution was discarded and the cells were washed three times with PBS prior to the addition of rhodamine phalloidin and DAPI staining solutions according to the manufacturer's instructions. The staining solution was removed and the cells were washed three times with PBS. Cells were left in PBS for fluorescence microscopy and photography.

### RNA extraction and cDNA synthesis

RNA was extracted from cells using acid guanidinium thiocyanate-phenol-chloroform extractions [22]. The integrity of the extracted RNA was verified via agarose gel electrophoresis. Intact, high quality RNA was indicated by the presence of the two, bright 28S and 18S rRNA bands in ethidium bromide stained agarose gels visualized under UV light. For each sample, approximately 1 μg of total RNA was treated with DNAse I (Sigma) to remove any residual DNA and converted to cDNA using the ImProm-II reverse transcription system (Promega) according to the manufacturer's instructions. Reactions were carried out in 20 μl volumes and all cDNA samples were diluted 1:5 in DNAse-free water prior to real-time PCR.

### Primers and qPCR

PCR primers for six candidate reference genes representing distinct functional classes were designed from DNA sequences available through the Entrez Nucleotide database (http://www.ncbi.nlm.nih.gov) (tables 1 and 2). The specificities of the primers were assessed by BLAST (http://www.ncbi.nlm.nih.gov), BLAT (genome.ucsd.edu) and oligoanalyzer 3.1 (http://www.idtdna.com) analyses. qPCR amplifications were performed in an iCycler iQ Real-Time PCR Detection System (Bio-Rad) using the iQ SYBR green supermix (Bio-Rad). Reactions were carried out in total volumes of 20 μl and included 250 nM of each primer and 2 μl of diluted cDNA template containing 100 ng cDNA. The thermal cycler conditions were as follows: Step 1, 95°C for 2:30 min; Step 2, 95°C for 10 s, 59°C for 10 s and 72°C for 25 s (45 cycles); step 3, melt curve analysis from 59-95°C in 0.5°C increments. The specificities of the PCR amplifications were assessed by the examination of the melt curves to confirm the presence of single gene-specific peaks. For the generation of standard curves, PCR products were purified through polyacrylamide gel electrophoresis. Resolved DNA bands were excised, crushed and eluted in 200 μl of pure water. Three-fold serial dilutions covering a 3-4 log dynamic range of the eluted PCR products was carried out and used as templates in real-time qPCR to generate standard curves.

### Data analysis

qPCR data in the form of cycle thresholds (CT) was exported to Microsoft Excel and SPSS. Box and whisker plots displaying the CT distributions were generated in SPSS. For geNorm analysis, raw CT data was converted to relative gene expression data using the (E+1)^-ΔCT ^transformation (where E is PCR efficiency). Gene stability values and the determination of the optimal number of control genes were elucidated by implementing the geNorm algorithm as previously described [21].

## Abbreviations

qPCR: Real-time quantitative reverse transcriptase PCR; CT: Cycle threshold; 18S: 18s Ribosomal RNA; ACTB: Beta-actin; B2M: Beta-2-microglobulin; GAPDH: Glyceraldehyde-3- phosphate dehydrogenase; HMBS: Hydroxymethylbilane synthase; HPRT1: Hypoxanthine guanine phosphoribosyl transferase 1;

## Competing interests

The authors declare that they have no competing interests.

## Authors' contributions

ASS designed and performed the experiments, analyzed and interpreted the data and wrote the manuscript. SRS assisted in designing the experiments and contributed to the critical review of the manuscript. NAM designed the study, contributed to the interpretation of the data and drafted the manuscript. All authors have read and approved the final manuscript.
